# STING agonists activate latently infected cells and enhance SIV-specific responses *ex vivo* in naturally SIV controlled cynomolgus macaques

**DOI:** 10.1038/s41598-019-42253-3

**Published:** 2019-04-11

**Authors:** Takuya Yamamoto, Tomohiro Kanuma, Shokichi Takahama, Tomotaka Okamura, Eiko Moriishi, Ken J. Ishii, Kazutaka Terahara, Yasuhiro Yasutomi

**Affiliations:** 1 Laboratory of Immunosenescence, National Institutes of Biomedical Innovation, Health and Nutrition, Osaka, 567-0085 Japan; 2 Center for AIDS Research, Kumamoto University, Kumamoto, 860-0811 Japan; 3 Tsukuba Primate Research Center, National Institutes of Biomedical Innovation, Health and Nutrition, Ibaraki, 305-0843 Japan; 4 Laboratory of Adjuvant Innovation, Center for Vaccine and Adjuvant Research, National Institutes of Biomedical Innovation, Health and Nutrition, Osaka, 567-0085 Japan; 5 Laboratory of Vaccine Science, World Premier International Immunology Frontier Research Center, Osaka University, Osaka, 565-0871 Japan; 6 Department of Immunology, National Institute of Infectious Diseases, Tokyo, 162-8640 Japan

## Abstract

To achieve a functional cure for HIV, treatment regimens that eradicate latently HIV-infected cells must be established. For this, many groups have attempted to reactivate latently-infected cells to induce cytopathic effects and/or elicit cytotoxic T lymphocyte (CTL)/NK cell-mediated immune responses to kill these cells. We believe that not only the reactivation of latently-infected cells, but also the induction of strong CTL responses, would be required for this. Here, we used typical immune activators that target pattern recognition receptors (PRRs). For our experimental model, we identified eight SIV-infected cynomolgus monkeys that became natural controllers of viremia. Although plasma viral loads were undetectable, we could measure SIV-DNA by qPCR in peripheral blood mononuclear cells (PBMCs). Using these PBMCs, we screened 10 distinct PRR ligands to measure IFN-α and IFN-γ production. Among these, STING ligands, cGAMP and c-di-AMP, and the TLR7/8 agonist R848 markedly increased cytokine levels. Both R848 and STING ligands could reactivate latently-infected cells in both cynomolgus monkeys and human PBMCs *in vitro*. Furthermore, c-di-AMP increased the frequency of SIV Gag-specific CD8^+^ T cells including polyfunctional CD8^+^ T cells, as compared to that in untreated control or R848-treated cells. Together, STING ligands might be candidates for HIV treatment.

## Introduction

The current hurdle for combination antiretroviral therapy (cART) against HIV-1 is that it is extremely difficult to eradicate latently-infected cells from the body^1^ despite controlling viral loads to undetectable levels in the plasma. To overcome this limitation of cART, some reports have indicated the importance of not only reactivating latently-infected cells, but also killing them via cytolytic immune cells such as cytolytic CD8^+^ T cells (CTLs) and natural killer (NK) cells^1,2^. Based on these data, theoretically, it might be possible to eradicate these latently-infected cells if those two responses can be induced simultaneously. However, to date, this has not been easily achieved. For example, the reactivation of latently-infected cells by latency reversing agents (LRAs) such as histone deacetylase inhibitors has been exploited for years, and one such reagent, namely SAHA, was found to reactivate these cells *in vitro* and *in vivo*^3^. However, this compound also caused defective CTL responses, which could be important for killing reactivated cells^4^. Thus, SAHA treatment itself could not eliminate latently-infected cells from the body.

Recently, some data have suggested that ligands for pattern recognition receptors (PRRs) might be potential immune stimulators that elicit strong immune responses and induce the reactivation of latently-infected cells^5–13^. Toll-like receptors (TLRs) are well-known PRRs and function as sensors of certain patterns to stimulate mainly innate immune signaling^14–16^. Accordingly, the TLR7 agonists GS-986 and GS-9620 have been evaluated as potential LRAs *in vitro* and *in vivo*^17–20^. However, there are other candidates such as TLR9 agonists that were also shown potently induce the reactivation of latently-infected cells^11,21^. The level of reactivation of latently-infected cells by either TLR7 or TLR9 agonists was further correlated with the level type I IFN upregulation, at least *in vitro*^22^. Moreover, this reactivation was compromised by an inhibitor of the type I IFN receptor or depletion of plasmacytoid dendritic cells (pDCs), which is a major source of type I IFN^23–25^. Therefore, type I IFN appears to be a key factor to evaluate the reactivation of infected cells. Additionally, IFN-γ is widely known as an important cytokine for CTL- and NK-cell-mediated immune responses. Thus, simultaneous induction of type I IFN and IFN-γ seems to lead to the simultaneous reactivation of latently-infected cells and enhanced CTL responses *in vitro*.

In this study, we first examined the immunostimulatory effects of 10 different ligands of different PRRs (summarized in Table 1). For this, we used simian immunodeficiency virus (SIV)-infected cynomolgus macaques that were aviremic and exhibited natural virus control as a model of latent infection. We studied peripheral blood mononuclear cells (PBMCs) from animals that were isolated before and 40 weeks after SIV infection, and found that the TLR7/8 agonist R848 and stimulator of interferon genes (STING) ligands, both c-di-AMP and 2′-3′-cGAMP, could dramatically upregulate levels of IFN-α and IFN-γ, and also Th1 type cytokine (IL-12p40) and MIP-1β, which have been reported to be inhibitory factors against HIV infection^26^, in the culture supernatant. Furthermore, we demonstrated that the number of SIV Gag DNA-positive cells was decreased and the expression levels of SIV Gag mRNA were increased in infected cells, and that SIV Gag-specific CTL responses were boosted with c-di-AMP stimulation. These findings, regarding the application of STING ligands, might form the basis for the clinical development of new immunostimulatory drugs that could provide a functional cure for HIV.

**Table 1:** Pattern recognition receptor (PRR) ligands used in this study and their target PRRs.

PRR ligands	Target PRRs	Cat. Number	Conc. for stimulation
Pam3CSK4	TLR1/2	InvivoGen vac-pms	10 μg/ml
Poly(I:C)	TLR3	InvivoGen vac-pic	10 μg/ml
LPS	TLR4	InvivoGen vac-3pelps	100 ng/ml
Flagellin	TLR5	InvivoGen vac-fla	10 μg/ml
R-848	TLR7/8	InvivoGen vac-r848	10 μg/ml
CpG ODN 2006	TLR9	InvivoGen vac-2006-1	10 μg/ml
Loxoribine	TLR7	InvivoGen tlrl-lox	10 μg/ml
TL8-506	TLR8	InvivoGen tlrl-tl8506	10 μg/ml
2′-3′-cGAMP	STING	InvivoGen vac-nacga23	10 μg/ml
c-di-AMP	STING	InvivoGen vac-nacda	10 μg/ml

## Results

### STING ligand and TLR7/8 ligands strongly activate innate immune responses in monkey PBMCs before and after SIV infection

**Figure 1:**
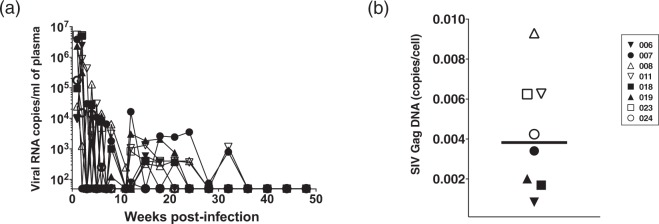
Characterization of model monkeys harbouring latently-infected cells. (**a**) Plasma viral loads in eight monkeys were plotted for 50 weeks. Viral loads were determined by qRT-PCR using plasma. Each data point for each monkey is depicted by a distinct shape. (**b**) Cellular SIV DNA in PBMCs from eight monkeys were plotted. PBMCs were obtained at 40 weeks post-infection. DNA copies were normalized and calculated based on cyno albumin DNA and synthetic SIV Gag cDNA. The bar indicates the median value.

In this study, we intrarectally challenged 30 monkeys with SIVmac239 and selected eight aviremic animals in which infection was naturally controlled after 40 weeks post-infection and over the following weeks (Fig. 1a). To examine viral reservoirs, we isolated PBMCs from each monkey at 40 weeks post-infection and measured SIV Gag DNA copy numbers by quantitative PCR. All eight monkeys were positive for SIV Gag DNA (Fig. 1b; range = 0.0008–0.009 and median = 0.004 copies/cells), suggesting that these monkeys harboured latently-infected cells. Thus, we utilized PBMCs from these monkeys as a model of latent infection for the following experiments.

**Figure 2:**
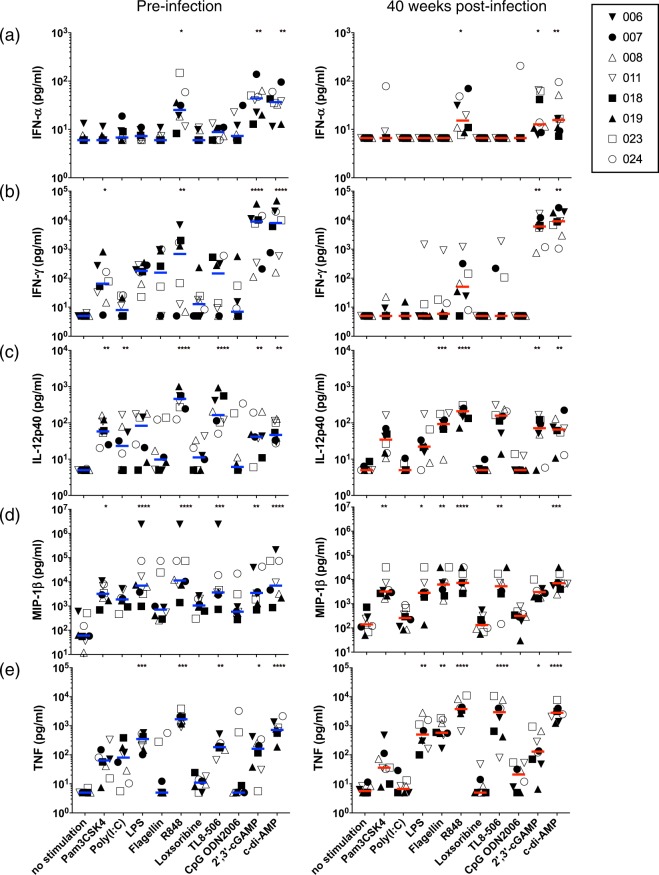
STING ligands robustly induce innate immune responses in SIV-infected PBMCs. Graphs show the effects of PAMP ligands on cytokine or chemokine levels in PBMC culture supernatants. PBMCs were isolated pre-infection and 40 weeks after SIV infection and were treated with PAMPs for 4 days; then, the levels of each cytokine/chemokine were measured in culture supernatants using a bio-plex platform. (**a**) Type I IFN (IFN-α) expression, (**b**) IFN-γ expression, (**c**) IL-12p40 expression, (**d**) MIP-1β expression, and (**e**) TNF expression in culture supernatants. Each data point from each monkey is depicted by a distinct shape, as in Fig. 1. Statistical significances were indicated by stars based on p-values (*P < 0.05, **P < 0.01, ***P < 0.001, ****P < 0.0001). Horizontal bars indicate median levels of each cytokine/chemokine.

First, we pre-screened 10 different PRR-related compounds that are well known to stimulate innate immunity (Supplementary Fig. S1 and Table 1). PBMCs were isolated from SIV natural controllers at pre- or 40 weeks post-infection, respectively. These cells were then stimulated with each compound with zidovudine (AZT) treatment for 24 hours. After collecting the culture supernatants, IFN-α, which is a type I IFN and reported to be a potential initiator of latently-infected cell activation^17^, was measured. As shown in Fig. 2a, IFN-α levels were significantly upregulated by a ligand of TLR7/8 (R848) and two ligands of STING (2′-3′-cGAMP and c-di-AMP) at both time points, as compared to those with no stimulation. Although the level of IFN-γ was upregulated by Pam3CSK4, R848, and STING ligands, compared to that with no stimulation, we could observe a significant increase in IFN-γ with STING ligands in SIV^+^ PBMCs (Fig. 2b).

Next, we assessed IL-12p40, MIP-1β, and TNF production. IL-12p40 is known to be involved in Th1 responses and is induced by type I IFN^27,28^ and MIP-1β, chemokines that are major factors produced by macrophages, and also known to be expressed in activated CTLs^29^. MIP-1β is also known to be expressed at higher levels in HIV-1 non-progressors than in progressors^29^. Furthermore, the activation of NF-κB is known to be involved in the reactivation of latently-infected cells^9,13^. When we used either SIV^−^ or SIV^+^ PBMCs, several compounds including STING ligands induced IL-12p40 and MIP-1β production compared to that with no stimulation; however, the strongest induction of these factors was observed with R848 stimulation for both SIV^−^ and SIV^+^ cases (Fig. 2c,d; P < 0.0001 for IL-12p40 and MIP-1β, respectively). In addition, the production of TNF was significantly increased by stimulating R848, 2′-3′-cGAMP, and c-di-AMP in latently-infected cells compared to that without stimulation (Fig. 2e).

Previously, it has been reported that the effect by STING ligands differs between human and murine cells^30^. Therefore, we examined whether the production of cytokines in healthy human or monkey PBMCs is different in response to STING ligand stimulation *in vitro*. As a result, the levels of cytokines produced in response to STING ligands in PBMCs were similar between human and cynomolgus monkey PBMCs (Supplementary Fig. S2).

Taken together, we concluded that R848 and STING ligands could be good candidates for not only inducing the reactivation of latently-infected cells but also eliciting anti-HIV immune responses, and these were used for further analyses.

### R848 and STING ligands reactivate latently-infected cells

**Figure 3:**
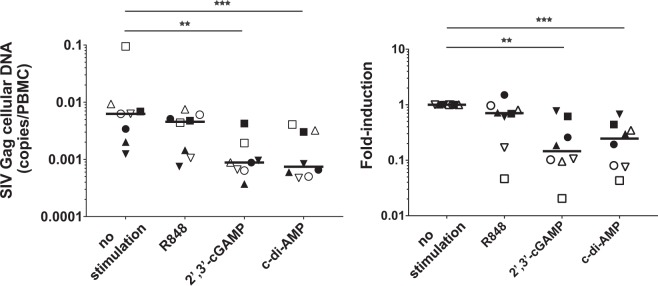
STING ligands decrease the amount of cellular SIV Gag cellular DNA/latently-infected cells. The effect of STING ligands and R848 on SIV Gag cellular DNA copy numbers is indicated. PBMCs isolated 40 weeks after SIV infection were treated with indicated ligands for 4 days; then, DNA from cells was analysed by qRT-PCR. (**a**) SIV Gag cellular DNA copies were normalized to PBMC numbers and plotted. (**b**) The fold-induction of SIV Gag cellular DNA copies/PBMC was plotted. Statistical significances were indicated by stars based on p-values (**P < 0.01, ***P < 0.001). Horizontal bars indicate median levels.

To examine the effect of R848 and two STING ligands (2′3′-cGAMP and c-d-AMP) on the latently-infected cells, we isolated PBMCs from monkeys exhibiting natural SIV control at 40 weeks post-infection and stimulated them with these ligands. After 4 days of cultivation in the presence of the anti-retroviral drug AZT, we measured copy numbers of SIV Gag cellular DNA in each monkey. We observed that either 2′-3′-cGAMP or c-di-AMP treatment, but not R848, could significantly decrease these copy numbers in PBMCs compared to those with no stimulation (Fig. 3a,b). These data imply that STING ligands could be useful to induce either cell death in infected cells, immune response-mediated killing in SIV-DNA-positive cells, or even both.

**Figure 4:**
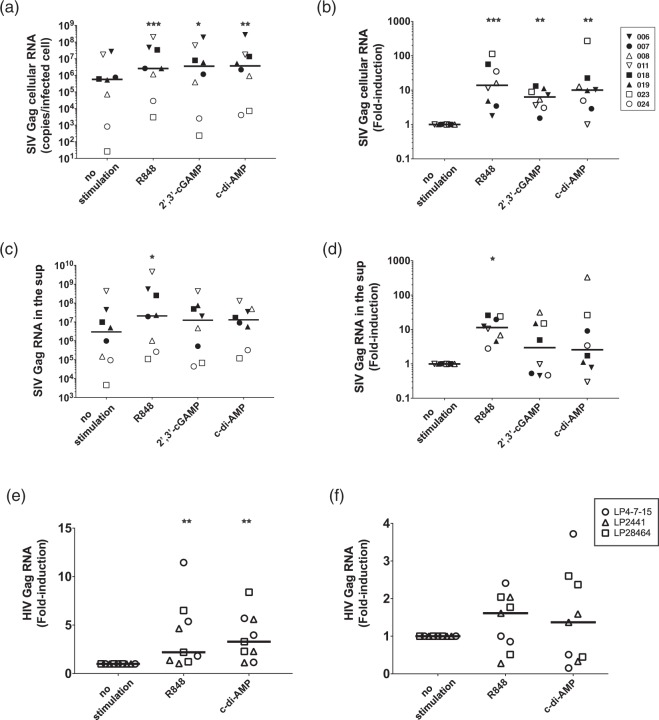
STING ligands increase the amount of cellular SIV-RNA/latently-infected cells. Effects of STING ligands and R848 on SIV Gag cellular/supernatant RNA copy numbers are indicated. PBMCs isolated 40 weeks after SIV-infection were treated with indicated ligands for 4 days; then, RNA from cells and supernatants were analysed by qRT-PCR. (**a**) SIV Gag cellular RNA copies normalized to infected cell numbers. (**b**) Fold-induction of SIV Gag cellular RNA copies/infected cell. (**c**) SIV Gag RNA copies in culture supernatant (sup) normalized to infected cell numbers. (**d**) Fold-induction of SIV Gag RNA copies in supernatant/infected cell. (**e**) HIV Gag cellular RNA copies normalized to infected EGFP^+^ cell numbers. (**f**) Fold-induction of HIV Gag cellular RNA copies/infected EGFP^+^ cell were plotted. Statistical significances were indicated by stars based on p-values (*P < 0.05, **P < 0.01, ***P < 0.001). Horizontal bars indicate median levels.

To further explore whether R848 and STING ligands could reactivate latently-infected cells, we estimated the production of SIV Gag RNA in infected cells by qRT-PCR. PBMCs were cultured for 4 days in the presence of each ligand plus AZT, and then levels of SIV Gag RNA in cells and culture supernatants were quantified (Supplementary Fig. S3a). All three compounds induced approximately 10-fold increases compared to copy numbers in unstimulated cells, and this was normalized to the number of the infected cells based on SIV Gag DNA copy numbers (Fig. 4a,b).

When we attempted to quantify SIV Gag RNA in culture supernatants, we were not able to distinguish the difference between high-level production of RNA by one cell or low-level RNA production by many cells. Thus, we simply quantified copy numbers from the same amount of culture supernatant in each well. We observed a significant increase in SIV Gag RNA in culture supernatants after only R848 stimulation, as compared to levels from unstimulated cells (Fig. 4c,d). We expected a similar increasing trend in the culture supernatants of both 2′-3′-cGAMP or c-di-AMP stimulated cells; however, we did not detect any significant differences compared to levels without stimulation. Since the number of infected cells was decreased by STING ligand stimulation, this might explain the lack of increase in SIV Gag RNA levels detected in the culture supernatants.

To further investigate whether these stimuli could reactivate human latently-infected cells, we utilized a primary CD4^+^ T cell model of HIV-1 latency established after activation through the T cell receptor and the subsequent return to quiescence, which was established previously by another group^31,32^. We cultured Bcl-2-expressing CD4^+^ T cells for 10 weeks to maintain quiescent infection (Supplementary Fig. S4a). Then, we infected these cells with VSV-pseudotyped HIV expressing EGFP^33^ and cultured them for another 10 weeks to return to quiescence. Using these resting CD4^+^ T cells that express HIV genes and EGFP, we cultured them with PBMCs (at 1000 EGFP^+^ cells to 1 × 10^6^ PBMCs) and stimulated them with either R848 or c-di-AMP. As a result, we observed a significant increase in HIV Gag cellular RNA with both stimulation conditions (Fig. 4e). Further, the reactivation of HIV latently-infected cells was not observed in the absence of autologous PBMCs (Fig. 4f; Supplementary Fig. S4b), suggesting that the reactivation of these CD4^+^ T cells is non-cell autonomous. Taken together, these results suggested that R848 and STING ligands can reactivate latently-infected cells in both cynomolgus monkey and human PBMCs *in vitro*.

### c-di-AMP enhances SIV-specific CTL responses

**Figure 5:**
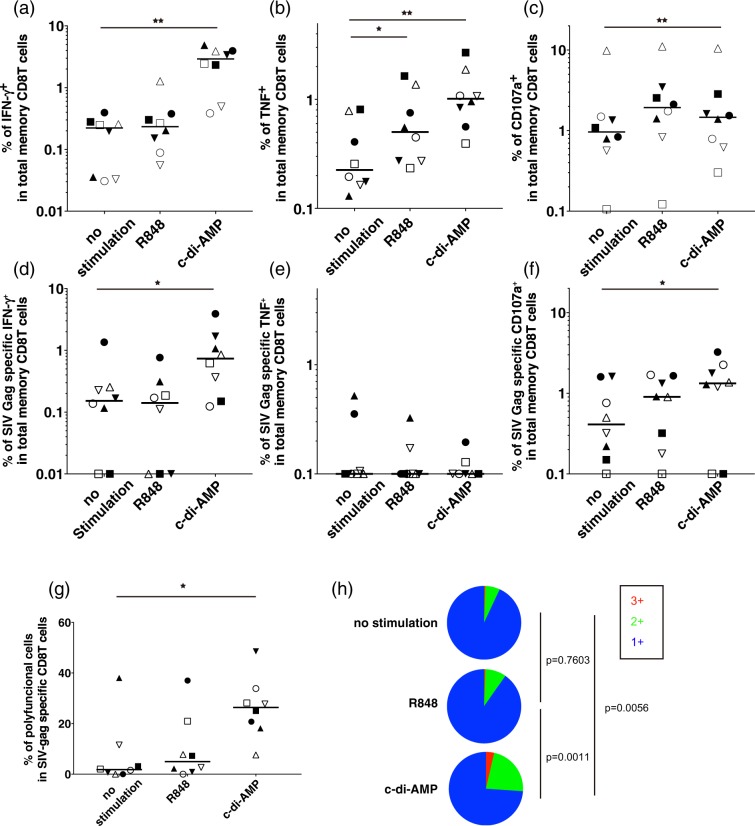
The effect of c-di-AMP and R848 on CTL responses as analysed by multicolour flow cytometry. (**a–c**) Percentages of IFN-γ^+^ (or TNF^+^, CD107a^+^) CD8^+^ T cells in the total memory CD8^+^ T cell population are plotted. (**d**,**f**) Percentages of SIV Gag-specific IFN-γ^+^ (or TNF^+^, CD107a^+^) CD8^+^ T cells among total memory CD8^+^ T cells. (**g**) Ratio of polyfunctional cells in SIV Gag-specific CD8^+^ T cells. Statistical significances were indicated by stars based on p-values (*P < 0.05, **P < 0.01). Horizontal bars indicate median levels. (**h**) The pie charts show the fraction of cells with one, two, and three functions. Statistical significances were indicated by permutation p-values.

Finally, we examined whether R848 and c-di-AMP could enhance SIV Gag-specific CTL responses. SIV^+^ PBMCs were stimulated with each adjuvant for 24 hours in the presence of AZT and then overlapping peptides corresponding to SIV Gag were added to the culture for another 6 hours. CD8^+^ T cells were analysed for the production IFN-γ and TNF, as well as the expression of CD107a, by flow cytometry (Supplementary Fig. S3b). The frequencies of IFN-γ-, TNF-, and CD107a-positive cells among total memory CD8 T cells were all significantly increased by c-di-AMP stimulation (Fig. 5a–c). However, with R848 stimulation, only the frequency of TNF-positive cells was increased. Regarding antigen-specific CD8^+^ T-cell responses, there were significant increases in IFN-γ production and CD107a expression, but not TNF production, in response to c-di-AMP together with TCR stimulation (Fig. 5d–g). Further, there were no changes in cytokine production and CD107a expression upon R848 and TCR stimulation. Moreover, we analysed the polyfunctionality of SIV Gag-specific CD8^+^ T-cell responses, which is defined by the positivity of more than two parameters in any parameter-positive cell (here, the three paprameters included IFN-γ, TNF, and CD107a). As shown in Fig. 5g,h, c-di-AMP with TCR stimulation boosted polyfunctional CD8^+^ T-cell responses compared to that with only TCR stimulation.

## Discussion

Models of SIV infection in non-human primates are well established and robust and possess many similarities to HIV infection in terms of disease progression. Using an optimized ART regimen against SIV, several groups have demonstrated consistent suppression of plasma viral loads in rhesus macaques, allowing these animals to be used as a translational animal model for HIV studies^34–36^. However, in all of these studies, cART treatment was initiated at an early phase of infection, and thus it is still unclear whether this acute cART model could mimic typical cART treatment in humans, which begins in the chronic phase of infection. In this study, we utilized PBMCs from cynomolgus macaques with naturally-controlled SIV infection as a model to investigate the characteristics of latent infection. In our hands, eight of the 30 cynomolgus macaques that were challenged by SIVmac239 became natural controllers of SIV infection. This could be because of differences in MHC- class I expression^37,38^, which restricts CTL responses. Although MHC-I alleles have been reported in cynomolgus macaques^39,40^, we did not determine their precise haplotypes in this study, in an effort to induce less bias regarding MHC haplotypes during our compound screening. We consider that this model might only be applicable to study latent infection in elite controllers and suggest that it is important to determine the MHC-I haplotypes of these monkeys in a future study. More importantly, we could not detect viral loads in these monkeys even though we could measure SIV Gag DNA in PBMCs, suggesting that this model does reflect some phenotypic aspects of latently-infected cells. Moreover, the *in vitro* models of latently-infected cell lines are very limited, and thus our model could be a complementary alternative to examine these cells, a possible mechanism to reverse latency, and the function of other LRAs *in vitro*.

In terms of the efficacy of inducing the reactivation of latently-infected cells, it has been reported that this is mediated by agonists of RIG-I and TLR7 through upregulation of type I IFN, at least *in vitro*^17,22^. This type I IFN induction was diminished by treatment with an inhibitor of type I IFN receptor or depletion of pDCs, which are a major source of type I IFN production^41^. These data indicate that type I IFN could be a useful bio-marker for predicting the reactivation of latently-infected cells upon stimulation. In addition to type I IFN, recent paper suggested that TNF is also involved in the TLR7-mediated reactivation of latently-infected cells^13^. In this study, we observed that both type I IFN and TNF were upregulated in response to not only R848 but also STING ligands, suggesting that these ligands possibly mediate the reactivation of latently-infected cells, at least *in vitro*. STING is a transmembrane protein located in the endoplasmic reticulum of many cell types including T-lymphocytes, macrophages, and DCs^42–44^. The function of STING is as a sensor for cytosolic cyclic-di-nucleotides, and it is involved in the expression of inflammatory cytokines and IFNs^42,45^. Indeed, we experimentally confirmed that R848 and STING ligands could reactivate latently-infected cells according to SIV Gag cellular RNA copy numbers in infected cells. This trend was similar to that observed in an *in vitro* HIV latency model (Fig. 3e). Although the upregulation SIV Gag RNA in the culture supernatant was only observed after R848 stimulation, this might be because STING ligands reduced the absolute numbers of latently-infected cells in culture, which might have affected the accumulation of SIV Gag RNA in the supernatant. Of note, it is considerable that induction of HIV/SIV RNA and reduction of HIV/SIV DNA does not often correlate with etiehr the magnitude of the reduction of the viral reservoirs or the amount of replication-competent viruses^46–52^. Since we only examined the levels of viral, cell-associated RNA and cell-associated DNA by standard qRT-PCR/qPCR, it would be important to further evaluate whether the treatment of STING ligand can reduce the actual size of latent reservoirs or replication-competent viruses *in vitro* by using different methods^53–55^.

Moreover, IFN-γ was upregulated by R848 and STING ligands suggesting that these ligands can enhance IFN-γ-related immune responses such as those mediated by CTLs and NK cells. Surprisingly, we observed that the STING ligand c-di-AMP, but not the TLR7/8 ligand R848, increased the frequency of SIV Gag-specific CD8 T-cell responses upon TCR stimulation. Furthermore, based on polyfunctional analysis, c-di-AMP treatment increased the proportion of polyfunctional cells in SIV Gag-specific CD8 T cells. It is widely known that the percentage of polyfunctional CTLs correlates well with anti-HIV-1 CTL responses^29^, suggesting that STING ligands could potentially improve the quality of SIV Gag-specific CD8 T cells to effectively kill the infected cells. This might also be related to the decrease in the number of SIV-infected cells, based on SIVGag DNA copy numbers, upon STING ligand treatment (Fig. 3).

Here, we demonstrated that both TLR7/8 ligand and STING ligands could increase the production of type I IFN and Th1 cytokines including IFN-γ in SIV^−^ and SIV^+^ PBMCs. However, principal component analysis (PCA) of the effects of PRR ligands on the levels of 12 cytokines revealed that STING ligands were clustered differently from R848, which indicates that these ligands have different effects on infected cells and immune cells (Supplementary Fig. S5). One potential reason for this could be the types of receptor-expressing cells. Although both TLR7/8 and STING signalling are known to trigger the expression of type I IFN and IFN-γ, STING is expressed ubiquitously in many cell types including epithelial cells, T-lymphocytes, macrophages, and DCs^42,44^; moreover, the expression of TLR7 is relatively specific for restricted cell populations. In this study, we actually observed different outcomes after R848 and c-di-AMP treatment. c-di-AMP increased SIV Gag cellular RNA in latently-infected cells and CTL responses in PBMCs isolated from animals with naturally-controlled SIV infection. In contrast, the TLR7/8 agonist R848 did not enhance SIV Gag-specific CTL responses, despite its ability to reverse latency. Delineating factors that affect these differences could be important to understand the underlying mechanism to efficiently enhance CTLs. Further study will be needed to address this point.

Another important factor for the eradication of latently-infected cells *in vivo* is the location of these cells and effector cells in the body. It has been gradually revealed that latently-infected cells accumulate in lymphoid tissues upon cART treatment^56–59^. Thus, to efficiently eliminate such cells from the entire body, we might need to deliver these agents to the lymph node. A recent paper reported that nanoparticalization of STING ligands could result in lymph node targeting^60^. Therefore, these types of approaches could allow for the more effective use of c-di-AMP *in vivo*.

Taken together, we utilized PBMCs from cynomolgus macaques with naturally-controlled SIV infection as a model of latent infection, and demonstrated the following: the STING ligand can 1) induce the production of type I IFN and Th1 cytokines including IFN-γ, 2) decrease the number of SIV Gag DNA-positive cells, 3) increase the expression levels of SIV Gag cellular RNA, and 4) boost SIV Gag-specific CTL responses. These data suggest that the STING ligand could simultaneously stimulate essential factors required for reactivation and killing to eradicate latently-infected cells during treatment for HIV.

## Material and Methods

### Animal experiments and PBMC sampling

In total, eight male cynomolgus macaques (*Macaca fascicularis*), all of which were negative for SIV, simian type D retrovirus, simian T-cell lymphotropic virus, simian foamy virus, Epstein–Barr virus, cytomegalovirus, and B virus were used. All were inoculated intrarectally with pathogenic SIVmac239 at 5 × 10^4^ TCID50 of SIVmac239. All animal studies were performed in TPRC, National Institutes of Biomedical Innovation, Health and Nutrition (NIBIOHN), after approval by the Committee on the Ethics of Animal Experiments of NIBIOHN, in accordance with the guidelines for animal experiments at NIBIOHN. The animals were used under the supervision of the veterinarians in charge of the animal facility.

Human PBMCs were isolated from healthy adult volunteers after obtaining the written informed consent from each individual. The protocol for collecting blood samples was approved by the Ethical Committee of NIBIOHN. The experimental protocols to use these samples were approved by the Ethical Committees and the Institutional Review Boards of NIBIOHN, Osaka University and National Institute of Infectious Diseases, respectively. All methods were performed in accordance with the approved guidelines and regulations.

All PBMCs and plasma isolation from blood samples containing EDTA, using Ficoll-Paque PLUS (GE Healthcare, Buckinghamshire, U.K.), was performed as previously described^61^. Either fresh or frozen PBMCs were placed in FBS containing 10% DMSO and stored in liquid-phase nitrogen until analysis. Complete blood counts were performed using an automated hematology analyzer (Sysmex K-4500; Sysmex, Hyogo, Japan).

### Reagents

All adjuvants in Table 1 were purchased from InvivoGen, CA. The peptides (15-mers overlapping by 11 residues) corresponding to the full-length SIVmac239 Gag peptide were obtained from the National Institutes of Health AIDS Research and Reference Reagent Program. AZT (CAS30516-87-1) was purchased from Tokyo Chemical Industry, Japan.

### Model of HIV latency

We synthesized cDNA encoding Bcl-2 (GenBank: EU287875.1) and cloned it into the pLVX-EF1a-IRES-mCherry Vector (Clontech, CA). For the preparation of Bcl-2-transduced lenti viruses, the Lenti-X HTX Packaging Systems (Clontech) were used. For the preparation of VSV-peudotyped HIV-1 (VSV-G-HIV-1_NL-EΔenv_), pVSV-G and pNL-EΔenv were used as described previously^62^. After 48–72 hours of transfection using 293T cells, the culture supernatants were collected and concentrated with the Lenti-X Concentrator (Clontech); HIV-1 p24 antigen was measured by an in-house enzyme-linked immunosorbent assay (ELISA)^63^. The infectivity was determined by either mCherry or EGFP expression in 293T cells.

Human primary CD4^+^ T cells were prepared with HIV^−^ PBMCs using the EasySep Human CD4^+^ T Cell Enrichment Kit (StemCell Technologies, Vancouver, BC, Canada). The primary CD4^+^ T cells were activated with 1 μg/ml of immobilized anti-CD3 monoclonal antibody (UCHT1; Thermo Fisher Scientific) plus 1 μg/ml of soluble anti-CD28 monoclonal antibody (CD28.2; Thermo Fisher Scientific) for 3 days in R-10 medium (RPMI-1640, 10% FBS, 100 U/ml penicillin, and 100 μg/ml streptomycin) supplemented with human AB serum and IL-2 (100 U/ml). Activated CD4^+^ T cells were infected with Bcl-2-transduced lentiviruses by spinoculation as described elsewhere^64^ and cultured in the presence of IL-2 for 3 days. Then, Bcl-2-transduced mCherry^+^ cells were purified by sorting and then cultured for another 17 days (10 days with IL-2 first and then 7 more days without IL-2). To expand Bcl-2-transduced mCherry^+^ cells, we repeated a series of these treatments (3 days for activation, 10 days with IL-2, and 7 more days without IL-2) for a total of three times. After these expansion processes, the cells were acclimated without IL-2 for 3 more weeks to diminish the activated state. Finally, we confirmed mCherry expression in the cells by flow cytometry. To produce resting CD4^+^ T cells harbouring latent EGFP^+^ HIV-1, we stimulated Bcl-2-transduced mCherry^+^ cells for 2 days and then infected them with VSV-G-HIV-1_NL-EΔenv_ by spinoculation. Infected cells were cultured for 10 more weeks without IL-2 to stabilize the resting condition.

### Quantitative RT-PCR and quantitative PCR

Levels of SIV infection were monitored by measuring plasma viral RNA loads with a highly-sensitive quantitative real-time RT-PCR approach as described previously^65,66^. Briefly, viral RNA was isolated from plasma using the MagNA PureCompact Nucleic Acid Isolation kit (Roche Diagnostics, Rotkreuz, Switzerland). Real-time RT-PCR was performed using a QuantiTec Probe RT-PCR kit (Qiagen, Germany) and a Light Cycler 480 thermocycler (Roche Diagnostics, Rotkreuz, Switzerland). The limit of detection was calculated to be 100 viral RNA copies/ml. For the purpose of assessing the reactivation of latently-infected cells, frozen PBMCs were thawed and then incubated in the presence of AZT (1 μM) and adjuvant (10 μg/ml) in R-10 medium for 4 days. Viral RNA in culture supernatant samples was prepared from the culture supernatant of PBMCs using a QIAamp Viral RNA Mini Kit (Qiagen, Germany) according to the manufacturer’s protocol. Cellular RNA samples were extracted from PBMCs using NucleoSpin RNA XS (MACHEREY-NAGEL, Germany) according to the manufacturer’s protocol. Using the culture supernatant, RNA, and cellular RNA samples, SIV Gag was detected by highly sensitive quantitative real-time RT-PCR. A DNA sample was extracted from PBMCs using a NucleoSpin RNA/DNA Buffer Set (MACHEREY-NAGEL, Germany) according to the manufacturer’s protocol. Using these DNA samples, the concentration of proviral DNA was determined by quantitative PCR. SIVmac239 gag was amplified with the probe 5′-FAM-TGTCCACCTGCCATTAAGTCCCGA-TAMRA-3′ (where FAM is 6-carboxyfluorescein and TAMRA is 6-carboxytetramethylrhodamine) and the primers 5′-GCAGAGGAGGAAATTACCCAGTAC-3′ and 5′-CAATTTTACCCAGGCATTTAATGTT-3′.

### Measurement of *in vitro* cytokine and chemokine levels

Fresh PBMCs isolated from monkeys at either day 0 or week 40 were cultured in the presence of each adjuvant (the concentration for each adjuvant is listed in Table 1) for 1 day, and then culture supernatants were collected and analysed by ProcartaPlex NHP immune assays (IFN-α, IFN-γ, IL-12p40, MIP-1β, and TNF; Thermo Fisher Scientific) on a Bio-Plex 200 device (Bio-Rad, CA). For PCA analysis, we measured additional cytokines/chemokines by ProcartaPlex NHP immune assays (CXCL13, GrzB, IL-10, IL-1β, IL-1RA, IL-2, IL-6, and TNF; Thermo Fisher Scientific). Using the entire dataset, PCA analysis was performed using the R-package “prcomp”.

### Polychromatic flow cytometry

The flow cytometric analysis of monkey PBMCs using a modified LSRII flow cytometer (BD Biosciences, MA) was described previously^67,68^. Briefly, frozen PBMCs were thawed and then incubated in the presence of AZT (1 μM) and adjuvant (Table 1) in R-10 medium for 12 hours. Next, SIV Gag overlapping peptides (2 μg/ml) and CD107a-BV421 (H4A3) were added and cells were incubated for 1 hour. Thereafter, monensin (0.7 mg/ml; BD Biosciences, MA) and brefeldin A (1 mg/ml; BD Biosciences, MA) were added and cells were incubated for 5 hours. Next, the cells were surface-stained with the following antibodies: CD95-PE-Cy5 (DX2; from BD Biosciences, MA); CD4-BV570 (OKT-4) and CD8-BV510 (SK1; from BioLegend, CA); CD28-ECD (CD28.2; Beckman Coulter, CA). LIVE/DEAD Fixable Violet and Blue Dead CellStain Kit (Life Technologies) were used to exclude dead cells from the analysis.

Following permeabilization (Cytofix/Cytoperm kit; BD Biosciences, MA), cells were stained with CD3-APC-Cy7 (SP34-2), IFN-γ–Alexa Fluor 488 (B27), TNF-PE-Cy7 (Mab11), and IL-2-APC (MQ1-17H12; all from BD Biosciences). For T cell analysis, cells were analysed using a modified LSRII flow cytometer (BD Biosciences, MA) as described previously^67^. Data were analysed using FlowJo software version 9.9.6 (Tree Star, OR). Polyfunctionality analysis was performed using SPICE version 5.35 (Mario Roederer, ImmunoTechnology Section, VRC/NIAID/NIH).

### Statistics

Experimental variables were analysed by performing a two-tailed unpaired t-test with Welch correction, a two-tailed paired t-test, Mann–Whitney U test, one-way ANOVA, and a two-tailed Wilcoxon matched-pairs signed-rank test. A p value < 0.05 was considered significant. GraphPad Prism statistical analysis software (Version 7; GraphPad Software, CA) was used throughout. Permutation p-values shown in Fig. 5h were caluculated by SPICE.

## Supplementary information

Supplemental Figures
