# Supplementary material for: STING agonists activate latently infected cells and enhance SIV-specific responses ex vivo in naturally SIV controlled cynomolgus macaques
Source: Sci Rep. 2019 Apr 11;9:5917. doi: 10.1038/s41598-019-42253-3 (PMC6459902; doi:10.1038/s41598-019-42253-3)
Supplement: Supplementary file 1 — Supplemental Figures [file 41598_2019_42253_MOESM1_ESM.pdf]

Supplementary Figures for *Scientific Reports*:

STING agonists activate latently infected cells and enhance SIV-specific responses *ex vivo* in naturally SIV controlled cynomolgus macaques

Takuya Yamamoto [1,2,7]* , Tomohiro Kanuma [1,3,7] , Shokichi Takahama [1,3] , Tomotaka Okamura [3] , Eiko Moriishi [1] , Ken J Ishii [4,5] , Kazutaka Terahara [6] , Yasuhiro Yasutomi[3]

1 Laboratory of Immunosenescence, National Institutes of Biomedical Innovation, Health and Nutrition, Osaka 567-0085, Japan.
2 Center for AIDS Research, Kumamoto University, Kumamoto 860-0811, Japan.
3 Tsukuba Primate Research Center, National Institutes of Biomedical Innovation, Health and Nutrition, Ibaraki 305-0843, Japan.
4 Laboratory of Adjuvant Innovation, Center for Vaccine and Adjuvant Research, National Institutes of Biomedical Innovation, Health and Nutrition, Osaka 567-0085, Japan
5 Laboratory of Vaccine Science, World Premier International Immunology Frontier Research Center, Osaka University, Osaka 565-0871, Japan
6 Department of Immunology, National Institute of Infectious Diseases, Tokyo 162-8640, Japan.
7 Contributed equally to this work.

*Correspondence author
E-mail: yamamotot2@nibiohn.go.jp
Tel:+81-72-641-9819
Fax:+81-72-641-9812

Supplementary Figure S1

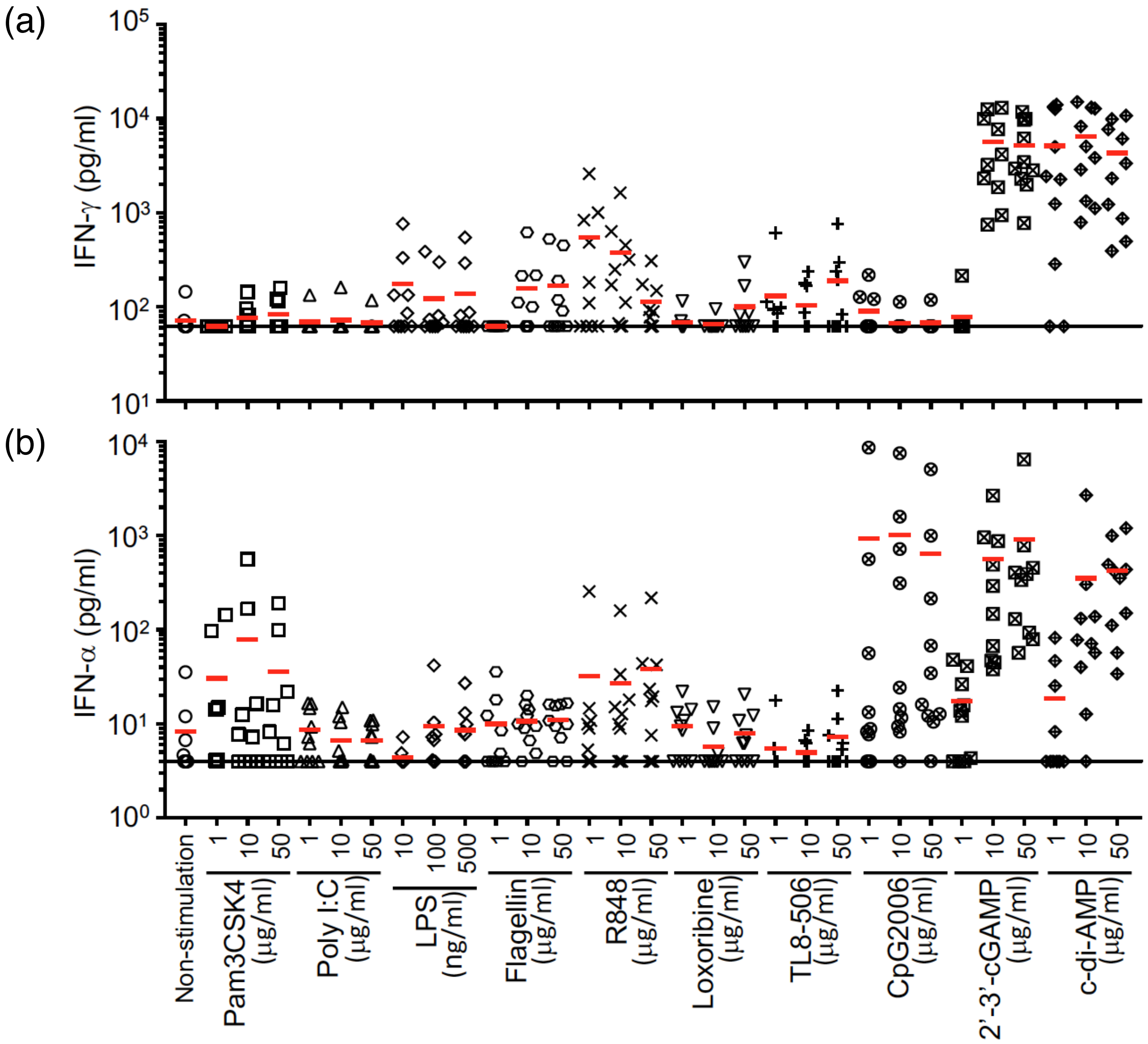

Optimization of adjuvant concentrations.
PBMCs isolated from cymonolgus monkeys were treated with indicated adjuvant for 24 hrs, then cytokines in culture supernatant were analyzed by ELISA. a) levels of IFN-γ were plotted. b) lebels of IFN-α were plotted. Bars indicate median values.

Supplementary Figure S2

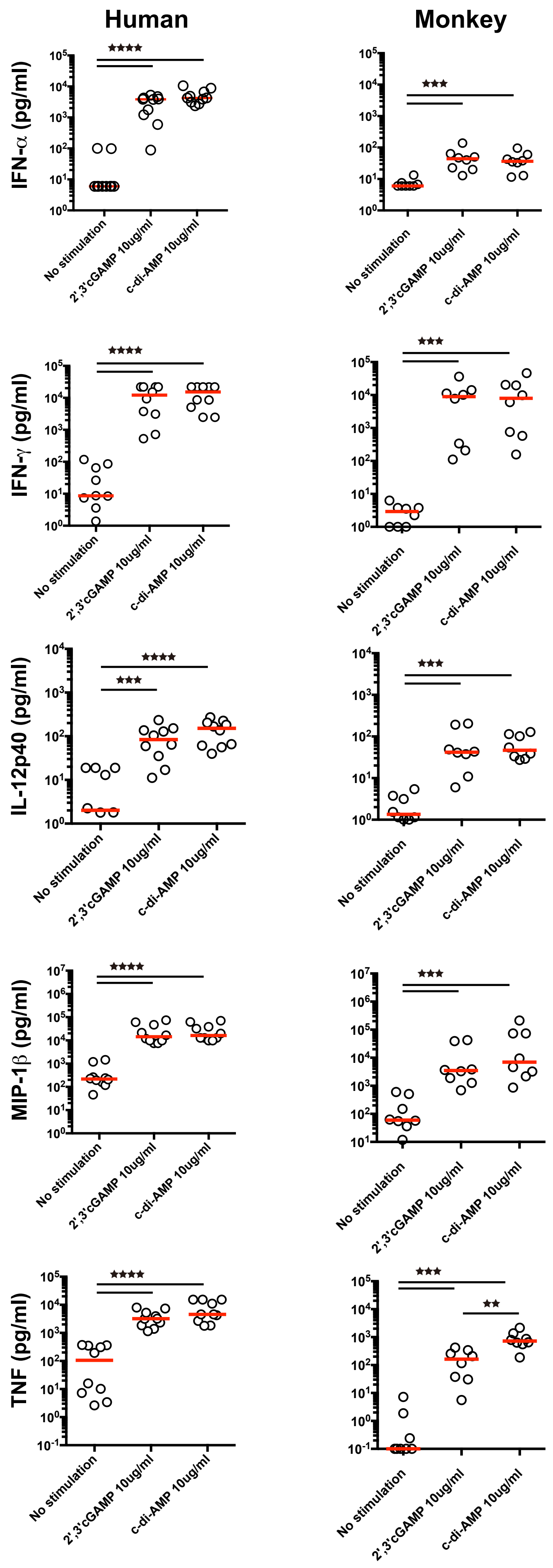

Comparison of the effect of STING ligands for human or monkey PBMCs.
PBMCs isolated from healthy human or cymonolgus monkeys were treated with indicated STING ligands for 24 hrs, then cytokines in culture supernatant were analyzed by ELISA. Bars indicate median values.

Supplementary Figure S3

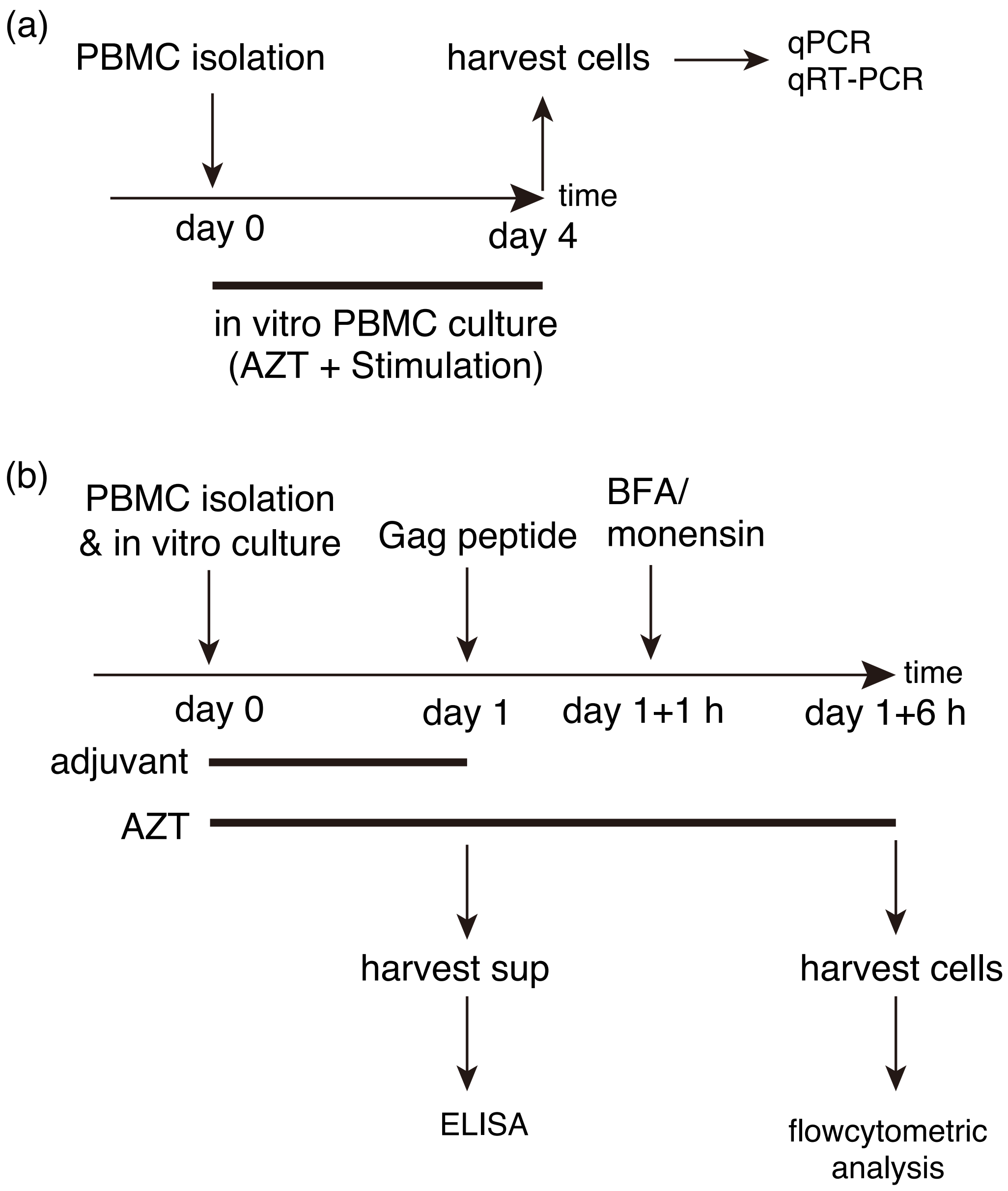

Schematic representation of experimental design.
(a) Evaluation of SIV Gag DNA or RNA in infected cells or culture supernatants. Isolated PBMCs were cultured with AZT and R848 or STING ligand for 4 days. After that, the cells were harvested, and the culture supernatants were collected, and then the cellular DNA or RNA and supernatant RNA were purified. These samples were evaluated by qPCR or qRT-PCR. (b) Identification of the function of adjuvant-stimulated PBMCs. Isolated PBMCs were cultured with AZT and adjuvants for 24 hours. After that, some cell culture supernatant was collected for the analysis of cytokine/chemokine expression by ELISA. These cells were stimulated with SIV Gag peptides for 24 hours and BFA and monensin were added after 1 hour. After SIV Gag peptide stimulation, the functions of these cells were evaluated by flow cytometry.

Supplementary Figure S4

(a)

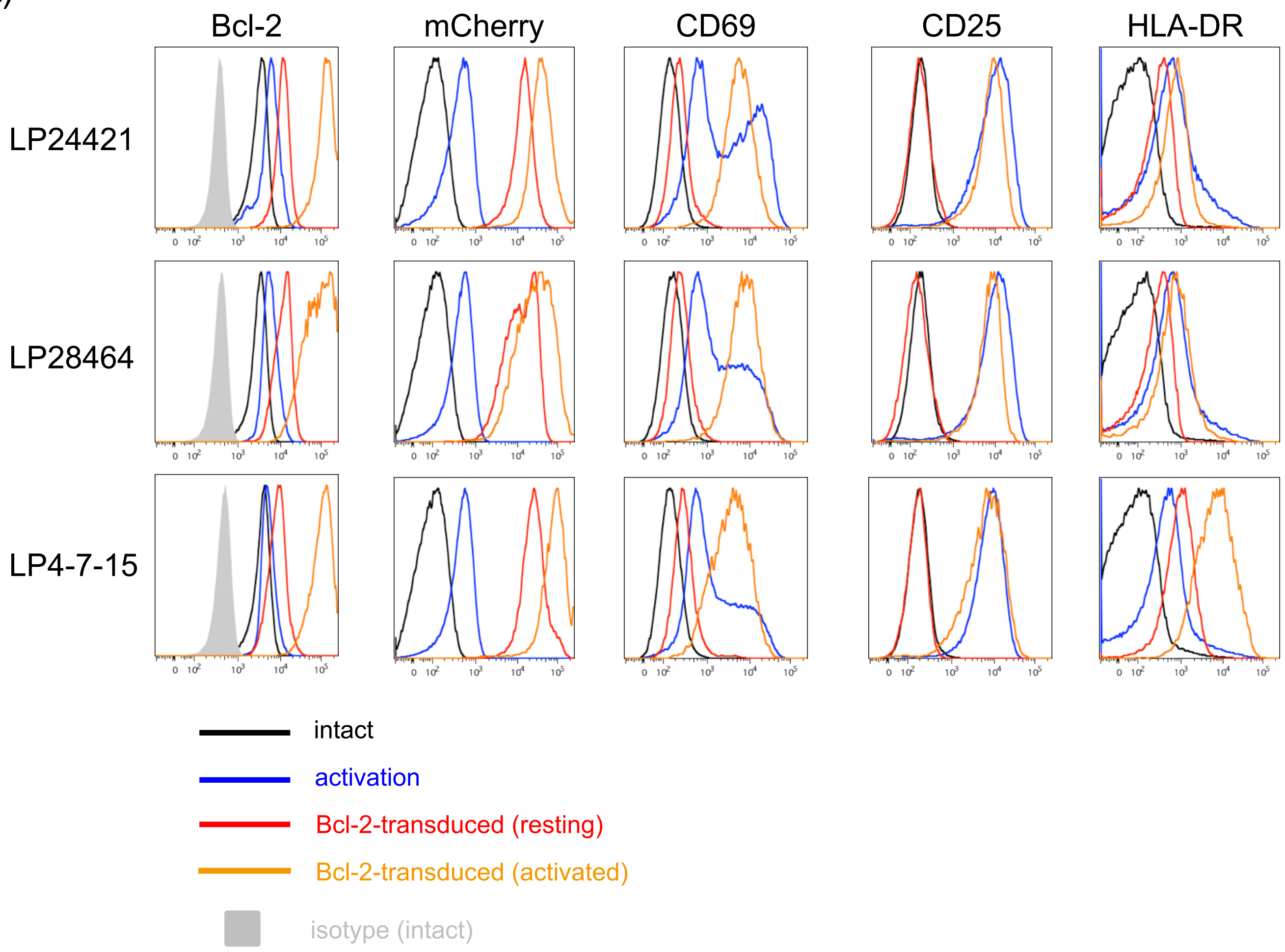

(b)

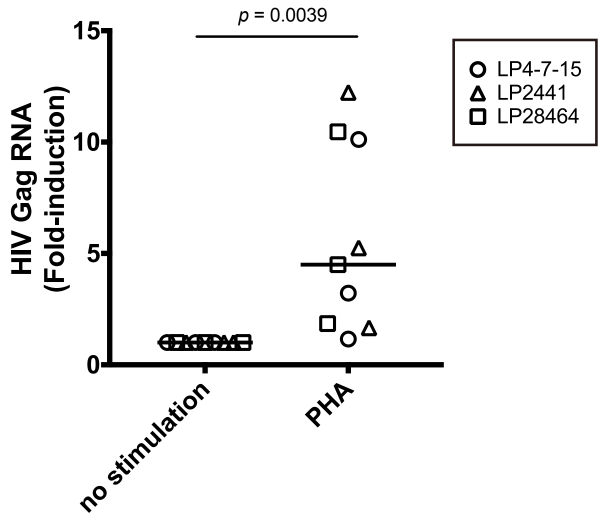

Establishment of HIV genes containing Bcl-2-transduced human $CD4^{+}$ T cells

a) Resting and activated status of parental and Bcl-2-expressing human $CD4^{+}$ T cells. $CD4^{+}$ cells isolated from PBMCs from patients were stimulated with CD3/CD28, and then cultured for 3 days. Then, cells were infected with Bcl-2/mCherry-expressing lentivirus and cultured for 3 days. mCherry-positive cells were sorted and expanded repeatedly for 10 weeks. Expression of Bcl-2/mCherry and activation markers in established Bcl-2-expressing $CD4^{+}$ cells were analysed by FACS.

b) Established $CD4^{+}$ T cells were stimulated with PHA for 4 days, then the levels of cellular HIV Gag RNA were analyzed by qPCR.

Supplementary Figure S5

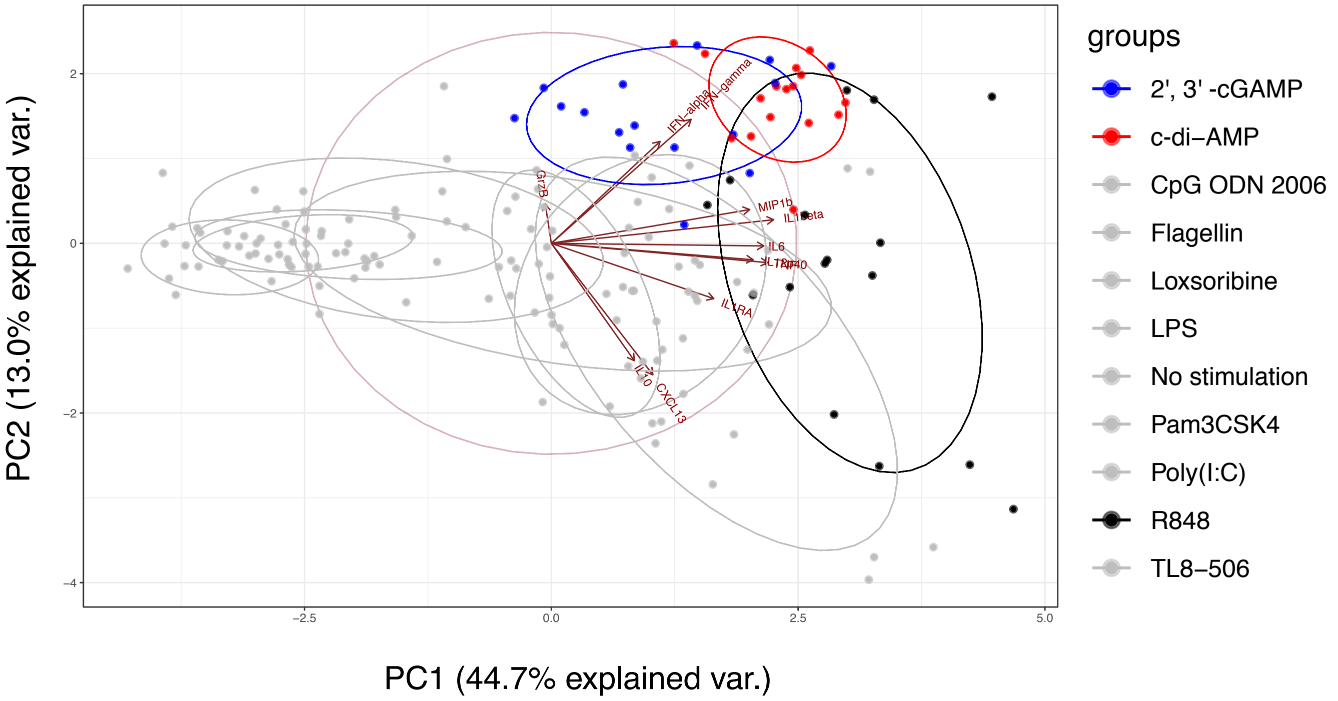

Plot showing the results of PCA analysis of the effect of each PAMP ligand on cytokine/chemokine expression. The levels of 12 cytokines/chemokines from eight monkeys were quantified by Bio-Plex, and then used as the source for PCA analysis. The X-axis indicates PC1, whereas the Y-axis indicates PC2 generated from 12 variables (= 12 cytokines/chemokines). Data were analysed by the R-package "prcomp", and then depicted by R-package "ggbiplot". Circles indicate the contribution of each PAMP ligand. Arrows point in the direction of the variables as projected into the 2-D plane. Circles indicate confidence ellipses.
